# Taurine supplementation decreases fat accumulation by suppressing FAS and enhancing ATGL through the ATGL pathway

**DOI:** 10.22038/ijbms.2024.76625.16590

**Published:** 2024

**Authors:** Xueying Geng, Yafang Feng, Congcong Yu, Yuyang Yao, Wen Chen, Junxia Guo, Yanzhen Zhang, Jing Zhang, Shengquan Mi

**Affiliations:** 1 College of Biochemical Engineering, Beijing Union University, Beijing, China; 2 Beijing Key Laboratory of Bioactive Substances and Functional Foods, Beijing Union University, Beijing, China

**Keywords:** Animal modelsmAtglistatin, High-fat diet, Lipid metabolism, Enzymes, Taurine

## Abstract

**Objective(s):**

Obesity leads to severe health issues like cardiovascular disease. Natural substances with anti-obesity properties are gaining attention. This study investigates the impact of taurine on lipid levels in rats fed a high-fat diet.

**Materials and Methods:**

The SD rats were fed a high-fat diet and treated with or without taurine for 21 weeks. Taurine was added to their drinking water, and an adipose triglyceride lipase (ATGL) inhibitor was injected for one week. The study evaluated the impact of taurine supplementation on the rats’ body weight, Lee index, body fat content, serum levels of triglycerides (TG), total cholesterol (TC), low-density lipoprotein-cholesterol (LDL-C), high-density lipoprotein-cholesterol (HDL-C), fatty acid synthase (FAS), ATGL, and peroxisome proliferator-activated receptor α (PPARα) in the liver. Fat accumulation in the liver and aortic arch was assessed through histopathological observations.

**Results:**

The study found that taurine reduced body weight, body fat, serum TG, TC, LDL-C levels, and lipid deposition in the liver and aortic arch while increasing serum HDL-C levels. Taurine intake also increased FAS and ATGL expression in the liver. Interestingly, the ATGL inhibitor atglistatin did not affect FAS and ATGL expression in the presence of taurine.

**Conclusion:**

Taurine can reduce fat deposition caused by a high-fat diet in SD rats by decreasing FAS content and increasing ATGL content. However, taurine does not fully regulate FAS and ATGL expression through the ATGL pathway.

## Introduction

WHO has found that the global increase in consumption of energy-dense foods and the sedentary nature of many forms of work is leading to a rise in the number of obese and overweight individuals. The 2030 Agenda for Sustainable Development recognizes NCDs (noncommunicable diseases) as a major challenge for sustainable development (1). Thus, natural substances with anti-obesity effects have attracted much interest from many researchers as potentially safe agents to reduce weight in the era of global obesity.

Taurine (2-aminoethanesulphonic acid) is a free amino acid that is found in high concentrations in almost all tissues in mammals. Although taurine is not involved in protein synthesis, it plays an important role in maintaining physiological function. Taurine contributes to numerous biological functions. For example, the anti-oxidant action reduces hypertension risks in rats (2), increases bile acid solubility by conjugating to bile acids (3), the anti-inflammatory effects have shown benefit to the cardiovascular system (4), taurine improves insulin resistance (5), and is also involved in energy metabolism in the muscle**,** adipose tissues, and the liver (6). Moreover, taurine has been shown to have an anti-obesity effect (7). It was reported that taurine supplementation (2% in drinking water) effectively resulted in the loss of fat mass in HFD-fed mice by using an LF50 body composition analyzer (8).

Research on the lipid-lowering effect of taurine mainly includes reducing TC (total cholesterol) content and reducing TG (triglyceride) content. A cell study showed that 20 mM taurine significantly reduced the TC content after treatment for 48 hr (9). Animal studies have shown that taurine attenuates the abnormal content of TC in the serum and liver of high-fat and high-cholesterol diet-fed rats and mice (10-11). In a study by Maleki *et al*., taurine supplementation (3000 mg/day) for eight weeks significantly decreased the level of TC in patients with T2DM (12). Compared with studies of taurine in reducing cholesterol, there are few reports on the metabolic mechanism of triglycerides. Taurine markedly reduced the higher concentration of cellular TG by preincubating with an oleic acid-rich medium (13). Kim *et al*. (14) used *Caenorhabditis elegans* as the experimental object and reported that taurine could reduce the TG of elegans in high-fat media. The content of total triglycerides in the liver of rats tended to be lower in the taurine-supplemented group than in the control group, but the lipid content was not significantly different between the two groups (15).

Therefore, we used SD rats as the research object to verify taurine biological activity on triglycerides. We focused on triglyceride degradation and the activities of various regulatory molecules in the catabolic pathway of triglyceride decomposition to further clarify the mechanism of taurine regulation on triglycerides.

## Materials and Methods

***Sources of animals and materials***

Male SD rats weighing between 200 g and 220 g were employed in this study. These rats were obtained from the Institute of Laboratory Animal Sciences, CAMS & PUMC (number: 114000500033042). Taurine (Tau, purity > 99.3 %) was a gift from Qianjiang Yongan Pharmaceutical Company. *Atglistatin* was purchased from TargetMol. Cremophor® EL was purchased from Sigma.

The TC, TG, SOD, and MDA assay kits were purchased from Nanjing Jiancheng Bioengineering Institute**;** the HDL-C and LDL-C kits were purchased from Zhongsheng North Control Biotechnology**;** and FAS, PPARα, and ATGL were purchased from Cusabio.

***Animals experiments***

The SD rats were raised in a single cave in an SPF animal facility (22±2 °C, 40%–55% relative humidity, 12-hour light and dark cycles). After adaptive feeding for one week, 80 rats were randomly divided into eight groups: N (normal feeding with normal drinking water group, n=16), NT (normal feeding with high-dose taurine drinking water group, n=16), HC (high-fat diet feeding with normal drinking water group, n=16), HCT1 (high-fat diet feeding with low-dose taurine drinking water group, n=16), HCT2 (high-fat diet feeding with high-dose taurine drinking water group, n=16), NY (normal feeding with *atglistatin* injected group, n=10), HCY (high-fat diet feeding with *atglistatin* injected group, n=10), HCT2Y (high-fat diet feeding and high dose taurine drinking water with *atglistatin* injected group, n=10).

***Reagents***

Low-dose taurine was given at 0.5 g/kg body weight, and high-dose taurine was given at 1 g/kg body weight. A dose of taurine was fully dissolved in normal water to obtain taurine water for each rat to drink from 5 pm to 9 am every day, and normal water was given during the rest of the day to drink freely. Generally, normal water was not given until the taurine water was finished. In addition, according to the average weight of rats in each group, taurine was adjusted weekly. After 17 weeks, the high dose of taurine increased to 2 g/kg. In the last week of the experiment, *atglistatin* was dissolved in PBS containing 0.25% Cremophor® EL and injected intraperitoneally at a dose of 200 µmol/kg. The rats were injected with *atglistatin* for seven days. Blinding was done by researchers, analysts, and animal caretakers to avoid any detection bias.

***Measurements***

The rats in each group were weighed weekly. At the end of the experiment, the body weight and the body length from the tip of the nose to the anus were measured. The net weight gain was calculated according to formula A, and the *Lee index* was calculated as formula B (16).

net weight gain=Final body weight-Initial body weight A

$\mathrm{Lee index}=\frac{\sqrt[{3}]{\mathrm{bodyweight}(g)}}{\mathrm{body lengh}(\mathrm{cm})}$ B

 The body fat content was measured with an EchoMRITM 500 Live Rats Body Composition Analyzer. Animals were sacrificed under pentobarbital sodium anesthesia, the whole blood was centrifuged after standing for half an hour at room temperature, and the serum was separated and packed into a small centrifuge tube. The left lobe livers of all rats were collected and preserved in liquid nitrogen immediately after the rat’s abdominal cavity was opened, and parts of the liver and aortic arch were fixed in formalin. The levels of serum TC, TG, LDL-C, and HDL-C were determined, and the levels of liver FAS, PPARα**,** and ATGL were quantified by ELISA. The histological sections of the liver and aortic arch were stained with hematoxylin-eosin to observe pathological changes. 

***Data analysis***

Statistical analysis was performed in this study using the SPSS software package (19.0). All measurement data are expressed as the mean ± SEM, and one-way ANOVA tests and Dunnett’s *post hoc* test were performed to compare the means of the different groups. A *P*-value less than 0.05 was considered statistically significant in all cases.

## Results

***Antiobesity effect of taurine in high-fat dietinduced obese SD rats***

**Figure 1:**
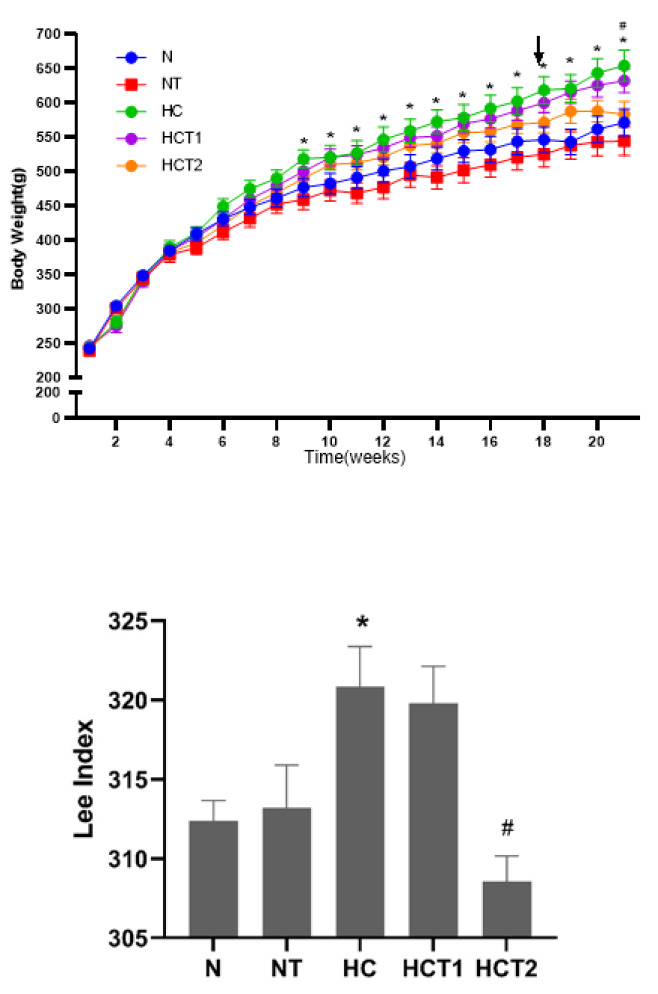
Effect of taurine on body weight loss in high-fat diet-fed SD rats ($\overset{\text{®}}{x}$ ±SE)

SD rats were fed a high-fat diet for 21 weeks to mimic human obesity. The high-fat diet over nine weeks significantly increased animal body weight compared to that of the rats receiving a normal diet (Figure 1A). At the 17^th^ week of feeding, there was no significant difference in body weight between the animals in the HC group, HCT1 group, and HCT2 group (*P*>0.05). Thus, the dose of taurine for the NT group and HCT2 group was increased to 2 g/kg, and the dose of taurine for the HCT1 group did not change. After 21 weeks of feeding, the means of the body weights (mean ± SEM) of animals in the HC and N groups were 654.3 ± 22.63 g and 571.4 ± 19.68 g, respectively. Long-term taurine supplementation (5% in drinking water) showed a significant trend of weight loss in the HCT2 group compared with that of the HC group (654.3 ± 22.63 g vs 583.4 ± 18.79 g) (*P*<0.05). However, there were no significant differences in body weight between the animals in the HC and HCT1 groups (654.3 ± 22.63 g vs 632.2 ± 17.31 g).

*Lee index* is one of the indicators used to describe the obesity status of adult rats. The larger the *Lee index*, the more obese the rats were. Taurine supplementation (5% in drinking water) showed an anti-obesity effect in high-fat diet-fed SD rats after 21 weeks of taurine feeding. Compared with that of the N group, the *Lee index* of the HC group was increased significantly (*P*<0.05) (Figure 1B). The *Lee index* of the HCT2 group was significantly lower than that of the HC group (*P*<0.05).

The fat, lean, free water and total water contents of rats were displayed by an EchoMRITM 500 Live Rats Body Composition Analyzer. The results are shown in Table 1. Body composition analysis showed that fat mass significantly increased, lean mass and total water showed a downward trend in the HC group, and the change in body composition in the HCT2 group was significantly reversed. Compared with that of the HC group, the fat mass decreased, and the lean mass and the total water increased in the HCT1 group, but the differences were not statistically significant. Taken together, the analysis of body weight, *Lee index*, and body composition suggested that taurine supplementation led to the loss of increased fat mass in high-fat diet-fed SD rats.

***Serum lipid concentrations***

Table 2 shows the effects of taurine on serum lipid concentrations in high-fat diet-fed rats. After 21 weeks of feeding, the serum TG in the HC group increased significantly compared to that of the N group. Compared with that in the HC group, TG in the HCT1 and HCT2 groups was markedly decreased with taurine supplementation, and similar trends existed in TC. The serum HDL-C was significantly lower than that expected in the HC group. However, the level of HDL-C was much higher only in the HCT2 group than in the HC group (*P*<0.05). Compared with that in the N group, LDL-C in the HC group was markedly increased, high-dose taurine diet-fed rats had lower LDL-C levels, and the difference was statistically significant (*P*<0.05).

***Histomorphology changes in the liver and aortic arch***

**Figure 2:**
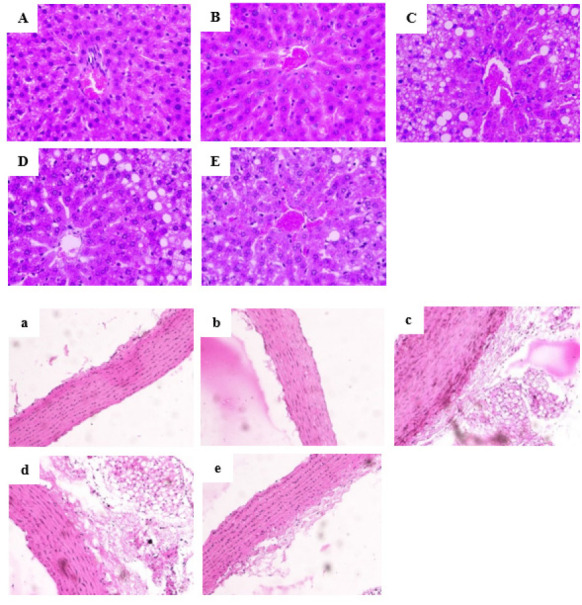
Histopathological observations of the liver (400X) (A-E) and aortic arch (200X) (a-e) sections from rats

Note: (A/a) N group; (B/b) NT group; (C/c) HC group; (D/d) HCT1 group; (E/e) HCT2 group. N: Compared with the normal diet group; HC: Compared with the high-fat model group, NT: Normal feeding with high-dose taurine drinking water group; HCT1: High-fat diet feeding with low-dose taurine drinking water group; HCT2: High-fat diet feeding with high-dose taurine drinking water group

The photomicrographs of the liver sections showed that in the normal group, the hepatocyte nucleus was large and round and located in the center of the cell. There were no fat vacuoles in the hepatic cells with neat arrangement. The hepatocyte status of the HC group was similar to that of the normal diet group (Figure 2B). The hepatocytes were swollen and loose and lost cellular boundaries, and the nuclei were squeezed away from the center of the cells because of many fat vacuoles in the HC group (Figure 2C). The hepatocyte arrangement in the HCT1 group was slightly improved, and the hepatocyte state was better than that in high-fat diet-fed animals, but there were still fat vacuoles and cell swelling (Figure 2D). In the HCT2 group, the hepatocytes were complete and orderly, the fat vacuoles were significantly reduced, and the cell morphology was close to normal.

The intimal surface of the aortic arch in the N group was smooth and flat, the edge boundary was clear, and there was no fat attachment layer on the inner wall. The aortic arch of the NT group was similar to that of the N group. However, the intima thickness of the aortic arch in the HC group was significantly increased compared with that in the N group. Moreover, there were many irregular fat foam attachments in the HC group. In the HCT1 group, there were a few fat foam attachments on the intima surface and intimal thickening. With no obvious intimal thickening, the fat foam cells in the intima in the HCT2 group were significantly decreased compared with those in the HC group.

A. From the 9^th^ week, the weight of the rats in the HC group was compared with that of the N group, and "*" means *P*<0.05. The arrow "↓" indicates that the dose of taurine in the HCT2 group was increased from week 17. AtIn the 21^st^ week, compared with the HC group, the HCT2 group had significantly lower body weight, with "#" indicating *P*<0.05

N: Compared with the normal diet group; HC: High-fat model group; NT: Normal feeding with high-dose taurine drinking water group; HCT1: High-fat diet feeding with low-dose taurine drinking water group; HCT2: High-fat diet feeding with high-dose taurine drinking water group

**Table 1:** Body fat content of rats ($\overset{\text{®}}{x}$±SE)

Groups	Fat（%）	Lean（%）	Free water（%）	Total water（%）
N	9.2±0.8	82.6±0.9	0.3±0.1	67.0±0.6
NT	12.8±1.5	79.6±1.7	0.2±0.0	64.0±1.3
HC	17.6±2.7*	75±2.6*	0.2±0.0	60.2±2.1*
HCT1	14.8±1.0	77.8±0.9	0.2±0.0	63.2±0.7
HCT2	12.2±1.9^#^	80.2±1.9^#^	0.2±0.0	65.0±1.7^#^

N: Compared with the normal diet group; HC: Compared with the high-fat model group, NT: Normal feeding with high-dose taurine drinking water group; HCT1: High-fat diet feeding with low-dose taurine drinking water group; HCT2: High-fat diet feeding with high-dose taurine drinking water group. “ “*” means *P*<0.05; #” means *P*<0.05

**Table 2:** Effects of taurine on blood lipid levels of rats ($\overset{\text{®}}{x}$±SE)

Content (mmol·L^-1^)	N	NT	HC	HCT1	HCT2
TG	0.38±0.06	0.37±0.05	0.6±0.05^*^	0.43±0.06^#^	0.39±0.05^#^
TC	1.6±0.15	1.63±0.15	2.24±0.12^*^	1.77±0.11^#^	1.61±0.13^#^
HDL-C	0.58±0.02	0.56±0.02	0.47±0.02^*^	0.52±0.03	0.54±0.02^#^
LDL-C	0.26±0.02	0.25±0.02	0.43±0.02^*^	0.4±0.02	0.32±0.02^#^

TG: Triglyceride; TC: Total cholesterol; LDL-C: Low-density lipoprotein-cholesterol; HDL-C: High-density lipoprotein-cholesterol; N: Compared with the normal diet group; HC: Compared with the high-fat model group, NT: Normal feeding with high-dose taurine drinking water group; HCT1: High-fat diet feeding with low-dose taurine drinking water group; HCT2: High-fat diet feeding with high-dose taurine drinking water group. “*” means *P*<0.05; “#” means *P*<0.05

**Figure 3:**
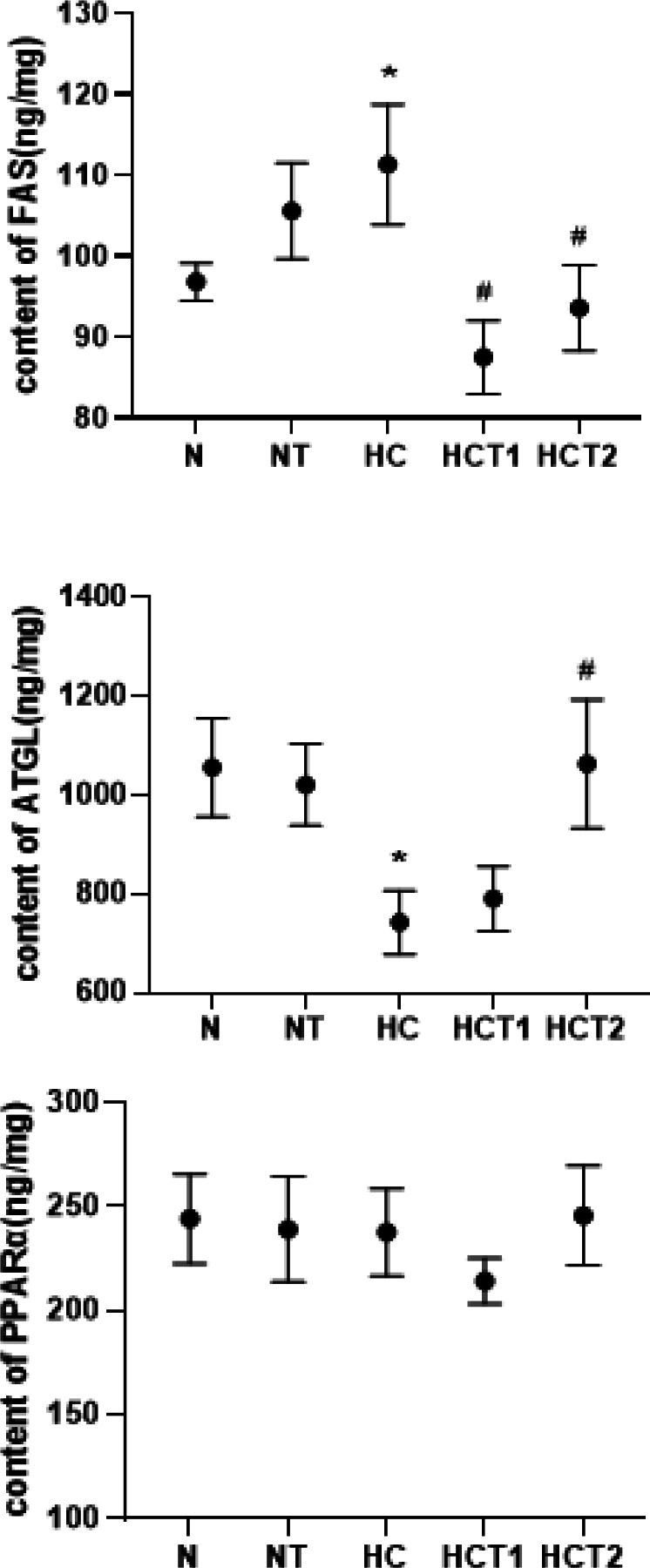
Effects of taurine on the contents of FAS and ATGL ($\overset{\text{®}}{x}$±SE)

"*": compared with the ordinary feed group (N), *P*<0.05; "#": compared with the high-fat model group (HC), *P*<0.05

FAS: Fatty acid synthase; ATGL: Adipose triglyceride lipase; N: Compared with the normal diet group; HC: Compared with the high-fat model group, NT: Normal feeding with high-dose taurine drinking water group; HCT1: High-fat diet feeding with low-dose taurine drinking water group; HCT2: High-fat diet feeding with high-dose taurine drinking water group

**Figure 4:**
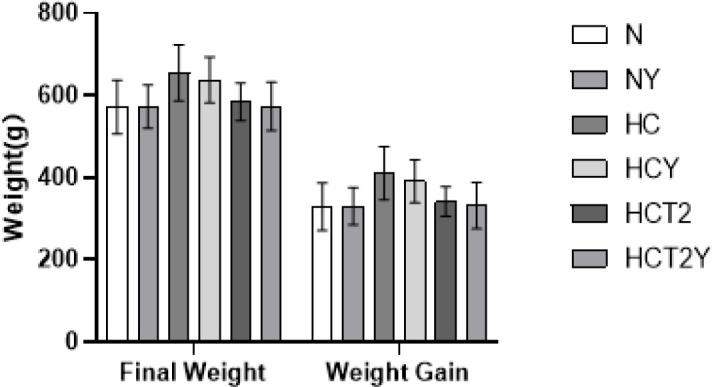
Effects of atglistatin on the body weight of rats ($\overset{\text{®}}{x}$±SE)

NY: Normal feeding with *atglistatin* injected group; HCY: High-fat diet feeding with *atglistatin* injected group; HCT2Y: High-fat diet feeding and high-dose taurine drinking water with of the *atglistatin*-injected group; HCT2: High-fat diet feeding with high-dose taurine drinking water group

**Table 3:** Effects of taurine on blood lipid levels after using atglistatin ($\overset{\text{®}}{x}$±SE)

Content (mmol·L^-1^)	N	NY	HC	HCY	HCT2Y	HCT2
TG	0.38±0.06	0.31±0.04	0.60±0.05	0.58±0.29	0.30±0.02	0.39±0.05
CHO	1.60±0.15	0.31±0.09	2.24±0.12	2.13±1.39	1.39±0.13	1.61±0.13
HDL-C	0.58±0.02	0.31±0.02	0.47±0.02	0.42±0.50	0.50±0.01	0.54±0.02
LDL-C	0.26±0.02	0.31±0.01	0.43±0.02	0.48±0.36	0.36±0.02	0.32±0.02

TG: Triglyceride; LDL-C: Low-density lipoprotein-cholesterol; HDL-C: High-density lipoprotein-cholesterol

**Figure 5:**
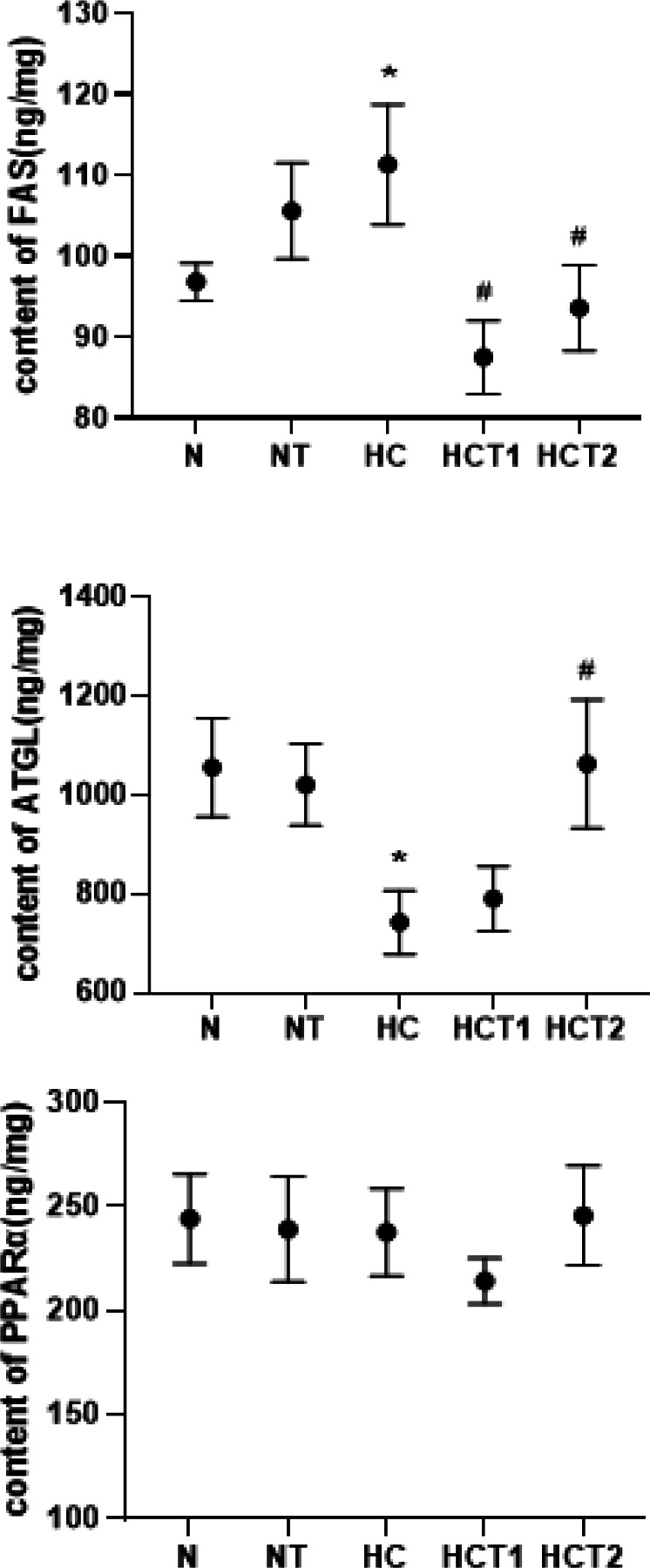
Contents of FAS, ATGL, and PPARα after *atglistatin* injection ($\overset{\text{®}}{x}$±SE)

FAS: Fatty acid synthase; ATGL: Adipose triglyceride lipase; N: Compared with the normal diet group; HC: Compared with the high-fat model group, NY: Normal feeding with *atglistatin* injected group; HCT2: High-fat diet feeding with high-dose taurine drinking water group; HCT2Y: High-fat diet feeding and high dose taurine drinking water with atglistatin injected group; PPARα: Peroxisome proliferator-activated receptor α

"*": compared with the normal feed group (n), *P*<0.05; "#": compared with the high-fat feed group (HC), *P*<0.05

***Effects of taurine on the expression of lipid metabolism enzymes***

To determine whether taurine induces lipid metabolism enzymes, the expression levels of FAS, ATGL**, **and PPARα were measured and compared (Figure 3). The results showed that high-fat diet feeding significantly increased the expression of FAS, and taurine supplementation significantly induced the down-regulation of FAS. As shown in Figure 4B, ATGL in the HC group was significantly decreased compared to that in the N group (*P*<0.05), and ATGL recovered in the HCT2 group (*P*<0.05). The level of PPARα did not show a significant difference among the five groups. It is suggested that taurine can reduce the FAS content and increase the ATGL content in high-fat diet-fed rats.

***Effects of atglistatin on body weight, serum lipids, and lipid metabolism enzymes of rats***

To verify that taurine plays a role in lipid-lowering through the ATGL pathway, the rats were injected with *atglistatin* for one week in our study. As shown in Figure 4, there was no significant difference in the final body weight or net weight gain between the NY and N groups or between the HCY and HC groups (*P*>0.05), suggesting that *atglistatin* had no significant effect on the body weight of the rats fed a normal diet or a high-fat diet. In addition, compared with that of the HCT2 group, the final body weight and net weight gain of the HCT2Y group had no significant change (*P*>0.05), indicating that *atglistatin* injection did not significantly change the effect of taurine supplementation.

Table 3 shows the effects of *atglistatin* on the serum lipid content of the rats in the various groups. There was no significant difference in TG, CHO, HDL-C, and LDL-C between the NY and N groups or between the HCY and HC groups (*P*>0.05), suggesting that *atglistatin* had no significant effect on the serum lipid level of the normal diet-fed and high-fat diet-fed rats. In addition, there was no significant difference in TG and CHO between the HCT2Y and HCT2 groups. The contents of HDL-C in the rats injected with *atglistatin* tended to be lower in the taurine-supplemented group than in the HCT2 group. Still, these contents were not significantly different between the two groups (*P*>0.05). The change in LDL-C was similar to that of HDL-C in the HCT2 and HCT2Y groups. After *atglistatin* injection, no significant changes were observed in serum lipids due to the inhibition of ATGL. The results of the comparison between the HCT2Y and HCT2 groups suggested that *atglistatin* did not completely disrupt the serum lipid-lowering effect of taurine, the function of taurine in regulating lipid metabolism was not affected by *atglistatin*, and its regulatory effect might be achieved in other ways.

As shown in Figure 5A, there was no significant difference in the content of FAS between the NY and N groups or between the HCY and HC groups (*P*>0.05), suggesting that *atglistatin* had no significant effect on the FAS level of normal diet-fed and high-fat diet-fed rats. There was also no significant difference in FAS content between the HCT2Y and HCT2 groups. In addition, *atglistatin* decreased the ATGL expression levels of normal diet-fed and high-fat diet-fed rats, and there was a significant difference between the NY and N groups (*P*<0.05). It is suggested that *atglistatin*s had effects on ATGL in normal diet-fed rats. The content of ATGL in the HCT2Y group was lower than that in the HCT2 group, but there was no significant difference (Figure 5B). There was no significant difference in the content of PPARα between the NY and N groups, between the HCY and HC groups, or between the HCT2Y and HCT2 groups (*P*>0.05). *Atglistatin* did not affect the PPARα content of the normal diet-fed rats, the high fat-fed rats, or the high fat-fed with taurine supplementation rats. Taurine could still reverse the contents of FAS and ATGL in the high-fat-fed rats after the ATGL pathway was blocked with *atglistatin*. These results indicated that ATGL was only one factor affecting lipid metabolism in high-fat diet-fed rats, such as TG decomposition acceleration, FAS content downregulation, and fatty acid synthesis reduction.

## Discussion

This study investigated whether taurine drinking water induces weight loss in high-fat diet-fed SD rats. SD rats were fed a normal diet, high-fat diet, or high-fat diet supplemented with high- and low-dose 5% taurine drinking water for 21 weeks. At the beginning of the experiment, no Significant difference in body weight was observed between the groups. At the end of the experiment, the rats fed a high-fat diet exhibited a significantly greater weight increase and fat accumulation than normal diet rats and developed obesity. Taurine supplementation had no effects on the normal diet-fed rats. In contrast, taurine supplementation had a positive association with the trends regarding body weight and *Lee index* in the high fat-fed rats. The results of the body composition measurement also showed that taurine drinking water significantly reduced the body fat content and increased the muscle tissue content when compared to that of the high-fat diet-fed but not taurine-supplemented rats. Our results are consistent with those of the Kim team’s research. They theorized that the potential anti-obesity effects of taurine might be partly due to thermogenic gene up-regulation in brown adipose tissue, such as UCP-1, Cox7a1**,** and Cox8b, and fat deposition down-regulation in inguinal white adipose tissue (17).

Moreover, taurine had a positive effect on reducing serum TG, TC**,** and LDL levels and increasing HDL levels in a high-fat diet-induced obesity rat model. According to our histopathological observations of the liver and aortic arch, taurine treatment decreased the liver’s fat content and the aortic arch’s intima thickness. A study showed that serum TC, TG**,** and LDL-C were higher, and HDL-C was lower in an alcoholic liver disease rat model than in normal rats, which could be significantly relieved by taurine administration (18).

Previous studies also showed that taurine could reduce serum lipid levels in rats fed a high-glucose and high-fat diet (-), which is consistent with our study’s results. Taurine’s antihyperlipidemic mechanisms are clearly complex, involving many enzymes in fat anabolism and catabolism, including FAS (21).

The liver is the main organ that metabolizes dietary fats, and the restriction of dietary cholesterol is beneficial for preventing liver fibrosis (22). The disorder of triglyceride metabolism is an important cause of fatty liver. In our study, there were obvious fat vacuoles in the livers of high-fat diet-fed rats, but taurine supplementation significantly reduced the number of fat vacuoles. FAS is one of the key enzymes in the liver’s fatty acid synthesis process. Its activity is to enhance and accelerate triglyceride synthesis. Chen (23) found that purslane water extract could significantly reduce the expression of FAS mRNA in the liver. The mRNA expression of FAS and PPARα, which can be significantly changed by ethanol, can also be regulated by taurine (18). Taurine also reduced the hepatic mRNA and protein levels of FAS in high-fat diet-fed rats (24). Our research suggested that a high-fat diet increased the content of FAS in the liver of SD rats, but daily intake of taurine may significantly reduce the FAS content. This finding is consistent with Lu *et al*. (2019), which showed taurine’s ability to decrease FAS levels in the livers of heat-exposed broilers (25). This result indicated that taurine might reduce the synthesis of triglycerides by down-regulating the content of FAS in the liver of obese rats.

Moreover, ATGL is the rate-limiting enzyme of triglyceride decomposition (26, 27) and a key enzyme for the release of FAS from TG. The regulation of ATGL is vital for maintaining a defined balance of lipids (28). Studies have shown that the expression of the ATGL gene in the adipose tissue of obese people is lower than that of normal people and that fat deposition may be related to the content of ATGL (29). The present research suggested that a high-fat diet significantly reduced the expression of ATGL in the livers of SD rats, but taurine significantly increased the expression of ATGL. The lncRNA-NEAT1 was found to modulate abnormal lipolysis by changing the expression of ATGL (30).

In addition, after 21 weeks of high-fat diet and taurine supplementation, there were no significant differences in the expression levels of PPARα in the liver of rats between the different groups in this study. This finding was consistent with the results of Kim *et al*. (2018), who found that taurine did not regulate the PPARα content of ICR mice fed a high-fat diet (7).

To verify whether taurine plays a role in lipid-lowering through the ATGL pathway, the contents of FAS, ATGL, and PPARα were detected after the injection of *atglistatin* in this study. After the rats were injected with *atglistatin*, there was no significant difference found in the body weight or the blood lipid level between the NY and N groups, and the situation between the HC and HCY groups and between the HCT2 and HCT2Y groups was similar. This result indicated that *atglistatin* did not affect the body weight or blood lipid content of either a normal diet or high-fat diet rats. In the present study, seven days of injection of *atglistatin* did not have a significant effect on the content of FAS and PPARα but significantly decreased the expression of ATGL in the liver, indicating that *atglistatin* reduced ATGL expression in normal diet rats. Moreover, the contents of FAS and PPARα had no obvious change in the HCY group compared with that of the HC group. Although *atglistatin* did not significantly change the ATGL level in the HC group, it tended to reduce the expression of ATGL in the liver of the rats in the HCY group.* Atglistatin* had no effect on the content of FAS and PPARα in the high-fat diet-fed SD rats treated with taurine, but it tended to reduce the expression of ATGL in the liver of the rats in the HCT2Y group. The results suggested that *atglistatin* did not completely block the lipid-lowering process of taurine and that dietary taurine supplementation could alleviate the decrease in ATGL in the liver of *atglistatin*-injected rats. Theoretically, after the injection of *atglistatin*, the ATGL-cAMP pathway is blocked to counteract the effect of taurine. In contrast, there was no significant difference between the FAS and ATGL levels between the HCT2Y and HCT2 groups, indicating that taurine reduced FAS and increased ATGL in other ways to promote lipid metabolism. *Atglistatin* did not regulate lipid metabolism through the ATGL-cAMP pathway, which may be caused by the insufficient action time of *atglistatin*.

## Conclusion

A high-fat diet increases fat content in SD rats. Taurine reduces fat accumulation by reducing the expression of FAS and increasing the expression of ATGL. *Atglistatin* did not regulate the expression of FAS, ATGL**,** or PPARα.

## Data Availability

The datasets used or analyzed during the current study are available from the corresponding author upon reasonable request.
